# Longitudinal evaluation of the impact of immunosuppressive regimen on immune responses to COVID-19 vaccination in kidney transplant recipients

**DOI:** 10.3389/fmed.2022.978764

**Published:** 2022-08-22

**Authors:** Aurélie Wiedemann, Céline Pellaton, Manon Dekeyser, Lydia Guillaumat, Marie Déchenaud, Corinne Krief, Christine Lacabaratz, Philippe Grimbert, Giuseppe Pantaleo, Yves Lévy, Antoine Durrbach

**Affiliations:** ^1^Vaccine Research Institute, Université Paris-Est Créteil, Faculté de Médecine, INSERM U955, Créteil, France; ^2^Service of Immunology and Allergy, Department of Medicine, Lausanne University Hospital, University of Lausanne, Lausanne, Switzerland; ^3^Department of Nephrology, Assistance Publique Hopitaux de Paris (APHP), Creteil, France; ^4^Paris-Saclay University, Gustave Roussy Institut, Institut National de la Santé et de la Recherche Médicale (INSERM) - Unité Mixte de Recherche (UMR) 1186, Integrative Tumor Immunology and Immunotherapy, Villejuif, France; ^5^Université Paris-Est Créteil, Faculté de Médecine, INSERM U955, Créteil, France; ^6^Swiss Vaccine Research Institute, Lausanne University Hospital, University of Lausanne, Lausanne, Switzerland; ^7^Groupe Henri-Mondor Albert-Chenevier, Service Immunologie Clinique, Assistance Publique-Hôpitaux de Paris, Créteil, France

**Keywords:** COVID-19, immunocompromised, immunosuppressive regimen, mRNA vaccine, immune responses

## Abstract

Immunocompromised patients have a high risk of death from SARS-CoV-2 infection. Vaccination with an mRNA vaccine may protect these patients against severe COVID-19. Several studies have evaluated the impact of immune-suppressive drug regimens on cellular and humoral responses to SARS-CoV-2 variants of concern in this context. We performed a prospective longitudinal study assessing specific humoral (binding and neutralizing antibodies against spike (S) and T-lymphocyte (cytokine secretion and polyfunctionality) immune responses to anti-COVID-19 vaccination with at least two doses of BNT162b2 mRNA vaccine in stable kidney transplant recipients (KTR) on calcineurin inhibitor (CNI)- or belatacept-based treatment regimens. Fifty-two KTR−31 receiving CNI and 21 receiving belatacept—were enrolled in this study. After two doses of vaccine, 46.9% of patients developed anti-S IgG. Anti-spike IgG antibodies were produced in only 21.4% of the patients in the belatacept group, vs. 83.3% of those in the CNI group. The Beta and Delta variants and, more importantly, the Omicron variant, were less well neutralized than the Wuhan strain. T-cell functions were also much weaker in the belatacept group than in the CNI group. Renal transplant patients have an impaired humoral response to BNT162b2 vaccination. Belatacept-based regimens severely weaken both humoral and cellular vaccine responses. Clinically, careful evaluations of at least binding IgG responses, and prophylactic or post-exposure strategies are strongly recommended for transplant recipients on belatacept-based regimens.

## Introduction

Patients with a compromised immune system are highly susceptible to SARS-CoV-2 infection and are among those most at risk of developing severe COVID-19, long-term complications, or fatal disease (1, 2). These individuals are also less likely to respond to COVID-19 vaccines (3–5) because of their immunodeficiency, immunosuppressive treatment or clinical condition (6, 7).

Seroconversion rates following SARS-CoV-2 mRNA vaccination remain lower in kidney transplant recipients (KTR) than in healthy immunocompetent individuals after one, two, or even three vaccine injections, as recommended by the French health authorities since April 2021 (4, 5). This third (booster) injection increased seroconversion rates, but the frequency and magnitude of anti-Spike IgG responses remains lower in immunocompromised individuals than in the general population (7). This is unsurprisingly, as several previous studies in patients with kidney failure reported weak responses to vaccination against flu or hepatitis B, leading to the adaptation of vaccination protocols and recommendations to increase vaccine dose or the number of booster doses (8–10). The efficacy of such strategies for SARS-CoV-2 mRNA vaccines is unclear, but the weaker response to these vaccines in immunocompromised individuals is a general observation across all COVID-19 vaccine platforms (7).

Decreasing vaccine-induced protective immunity against SARS-CoV-2 variants of concern (VOCs) is another issue, with higher transmissibility and hospitalized breakthrough case rates among immunocompromised individuals (11). Moreover, vaccine-elicited anti-Spike (anti-S) IgG levels gradually decline over about 6 months in the general population, but much more rapidly immunocompromised individuals with solid cancers, inflammatory diseases, or after organ transplantation (6, 12–16).

The impact of immunosuppressive regimens on these cellular and humoral responses also warrants further investigation. Rates of seroconversion after COVID-19 vaccination are generally low in KTR treated with calcineurin inhibitors (CNI) or belatacept (CTLA-4 Ig), but with significant differences between these two regimen types (16–19). It remains unclear whether post-vaccination cellular immunity can compensate for waning immunity and/or immune escape by VOC, and few exploratory studies have evaluated cellular responses to vaccination in transplant patients (16). The different modes of action of CNI and belatacept may result in different impacts on cellular and humoral responses to vaccines. Belatacept is associated with higher rates of graft and patient survival after kidney transplantation, but can affect the balance between cellular (increasing the risk of T-cell rejection) and humoral (decreasing the risk of anti-HLA antibody production) rejection (20–24). It is therefore important to investigate the impact of these drugs on both cellular and humoral responses to vaccines. We characterized humoral responses by measuring levels of both binding and neutralizing IgG antibodies against the original, SARS-CoV-2 (2019-nCoV) and the Alpha, Beta, Gamma, Delta and Omicron variants, and functional T-cell responses to S peptides over a 6-month period in a prospective cohort of KTR treated with either CNI or belatacept.

## Materials and methods

### Patients

We enrolled 52 KTR in the VACOTRARE longitudinal single-center cohort, to evaluate the durability of immunity after COVID-19 mRNA vaccination. Patients received two injections of the BNT162b2 mRNA vaccine 4 weeks apart. At vaccination, 31 KTR were treated with CNI, and 21 were treated with belatacept (Table 1). During follow-up, 40 patients received a third injection (booster) of BNT162b2 mRNA vaccine in accordance with French national recommendations. Blood samples were collected before vaccination (pre-vacc), 15 days after the first dose (post-dose 1), 15 days after the second dose (post-dose 2), and then at months 3 (M3) and 6 (M6), for peripheral blood mononuclear cell (PBMC) and serum isolation. All adverse effects of vaccination and cases of viral infection were recorded. All patients gave written informed consent for the collection, storage, and use of biological samples. The study protocol was approved by the appropriate Ethics Committee (No. IDRCB: 2018-A01610-55).

**Table 1:** Patient characteristics.

	**All patients**	**CNI**	**Belatacept**	***P*-value**
Age (years) mean ± SD	49.7 ± 14.2	48.8 ± 14.2	51.1 ± 14.4	0.57
Sex (female) %	40.7	48.5	28.6	0.14
BMI (kg/m^2^) mean ± SD	27.6 ± 6.1	28.3 ± 6.4	26.5 ± 5.6	0.29
**Nephropathy (%)**				0.12
Glomerular	52.8	46.9	61.9	
Vascular	5.7	3.1	9.5	
Interstitial or APKD	30.2	40.6	14.3	
Other	11.3	9.4	14.3	
Transplantation rank > 1 (%)	19.2	22.6	14.3	0.33
Time since transplantation (years) mean ± SD	6.4 ± 5.2	6.6 ± 6.3	6.2 ± 3.2	0.80
**Risk factors at vaccination (%)**
Hypertension	88.5	87	90.4	0.70
Diabetes mellitus	17.3	16.1	19	0.78
Cardiovascular disease	3.8	0	10	0.053
Tumors	7.7	9.7	4.8	0.5
**Induction (%)**				0.59
rIL2 mAb	37.5	40.7	33.3	
Thymoglobulin	62.5	59.2	66.6	
**Immunosuppression**
CNI (%)	59.6	100	NA	NA
Belatacept (%)	40.4	NA	100	NA
Anti-metabolites (%)	63.5	61.3	71.4	0.50
mTOR inhibitors (%)	25	25.8	23.8	0.97
Steroids (%)	94	93.5	95.2	0.85
**Immunosuppressor dose mean** **±SD**
T0 tacrolimus (ng/ml)	5.2 ± 1.5	5.2 ± 1.5	NA	NA
T0 cyclosporine A (ng/ml)	139 ± 56	139 ± 56	NA	NA
MMF dose (mg/day)	956 ± 355	1,014 ± 349	885 ± 362	0.33
T0 certican (ng/ml)	4.5 ± 1	4.5 ± 1	4.5 ± 1	1
Steroid dose (mg/day)	5 ± 1.1	5 ± 1.1	5.1 ± 1.3	1
eGFR (ml/min)	46.4 ± 26	50.5 ± 24.8	40.3 ± 27.3	0.17
Lymphocytes (count/mm^3^) mean ± SD	1,398 ± 753	1,554 ± 811	1,190 ± 626	0.094
CD4	594 ± 384	660 ± 463	500 ± 210	0.23
CD8	538 ± 318	586 ± 635	472 ± 233	0.23
CD19	170 ± 173	121 ± 113		
Death (*n*)	2	2	0	0.23
COVID (*n*)	15	8	7	0.47

APKD, Autosomic polycystic kidney disease; BMI, Body Mass Index; CD, cluster of differentiation; CNI, Calcineurin Inhibitors; eGFR, estimated glomerular filtration rate according to the CDK-EPI formula; mTOR, mammalian target of rapamycin; NA, not applicable; rIL2 mAb, monoclonal antibody against interleukin 2 receptor; SD, Standard deviation; T0, trough level.

### Determinations of anti-spike IgG levels and VOC neutralization

The levels of binding and neutralizing IgG antibodies against Spike were determined in two Luminex-based assays (Luminex Corporation), as previously described (6, 25, 26). For binding assays, two different types of MagPlex beads (Luminex Corporation) were covalently coupled with the SARS-CoV-2 Spike protein trimer and nucleocapsid (NuC) protein. Beads were then incubated 1 h with a 1/300 serum dilution or a negative control of pre-pandemic pool of human AB serum (BioWest, VWR). After washing, we added anti-human IgG-PE (phycoerythrin) secondary antibody (One Lambda, Thermo Fisher Scientific) to each well and incubated the plate for 45 min. The plate was read with a Luminex 200 analyzer (Luminex Corporation, BioRad). The mean fluorescence intensity (MFI) signal of each serum was divided by the mean negative control MFI, to obtain a ratio. A conversion factor was applied to the ratio to obtain equivalent anti-S U/ml. For the Spike protein ACE2 surrogate neutralization assay, different types of MagPlex beads were covalently coupled with different spike variants (2019nCoV, Alpha, Beta, Gamma, Delta, Omicron) were incubated 1 h with serum samples at different dilutions (1:10; 1:30; 1:90; 1:270; 1:810; 1:2,430) in PBS. The negative control was as previously described. The positive control was a recombinant anti-Spike neutralizing antibody (CHUV, Lausanne). We added ACE2 mouse Fc fusion protein (EPFL, Lausanne) to the wells at a final concentration of 1 μg/ml. The plates were washed twice with PBS-Tween and anti-mouse IgG-PE secondary antibody (Invitrogen, Thermo Fisher Scientific) was added and the plates were read with a Luminex 200 analyzer (Luminex Corporation, BioRad). The negative control MFI was considered to correspond to 100% binding of the ACE2 receptor to the bead-coupled spike trimer. The MFI of the well containing the highest concentration (>1 μg/ml) of commercial anti-spike blocking antibody was considered to represent maximum inhibition. The percent inhibition of the spike protein trimer–ACE2 interaction was calculated as follows: % inhibition = (1 – ([MFI test dilution – MFI max inhibition]/[MFI max binding – MFI max inhibition]) × 100).

### Characterization of spike-specific T-cell responses

Briefly, PBMCs were stimulated by overnight incubation with two pools of 15-mer peptides, overlapping by 11 amino acids, covering the whole spike protein of the SARS-CoV-2 reference strain Human 2019-nCoV HKU-SZ-005b (JPT Peptide Technologies, Berlin, Germany): S1 (168 peptides), S2 (144 peptides). Total S-specific responses were determined by summing S1 and S2 responses. Unstimulated cells were used as a negative control. The flow cytometry panel included a viability marker, CD3, CD4, and CD8 for T-cell lineage determination, and antibodies against IFN-γ, IL-2, and TNF. Data were acquired on a LSRII Fortessa 4-laser (488, 640, 561, and 405 nm) cytometer (BD Biosciences), and analyzed with FlowJo software version 9.9.6 (Tree Star Inc.).

### Statistical analyses

Graphpad Prism software version 8 was used for non-linear four-parameter curve-fitting analyses, and for non-parametric statistics and plots, as described in the figure legends.

## Results

Fifty-two patients were enrolled in the VACOTRARE cohort (Table 1). Mean ± SD follow-up since kidney transplantation was 6.4 ± 5.2 years. Mean age ± SD was 49.7 ± 14.2 years, and 40.7% of the patients were female. Thirty-one patients were treated with CNI, and 21 with belatacept. In the CNI group, 8/31 patients were treated with a CNI-mTOR inhibitor (mTORi) combination and 23/31 were treated with a CNI-anti-metabolite (MMF or azathioprine) combination. Belatacept was associated with MMF or azathioprine in 15 patients, and with mTORi in five patients. All but two patients received low doses of steroids. The trough levels of CNI and mTORi at the time of vaccination are shown in Table 1. Renal function, age and lymphocyte counts were similar in the CNI and belatacept groups (Table 1). All patients received at least two doses of BTN162b2 mRNA vaccine, and 40 received a booster. This third dose was not administered to patients who died (*n* = 2), or experienced symptomatic COVID-19 during follow-up (*n* = 7) or to patients refusing the booster (*n* = 3).

**Figure 1:**
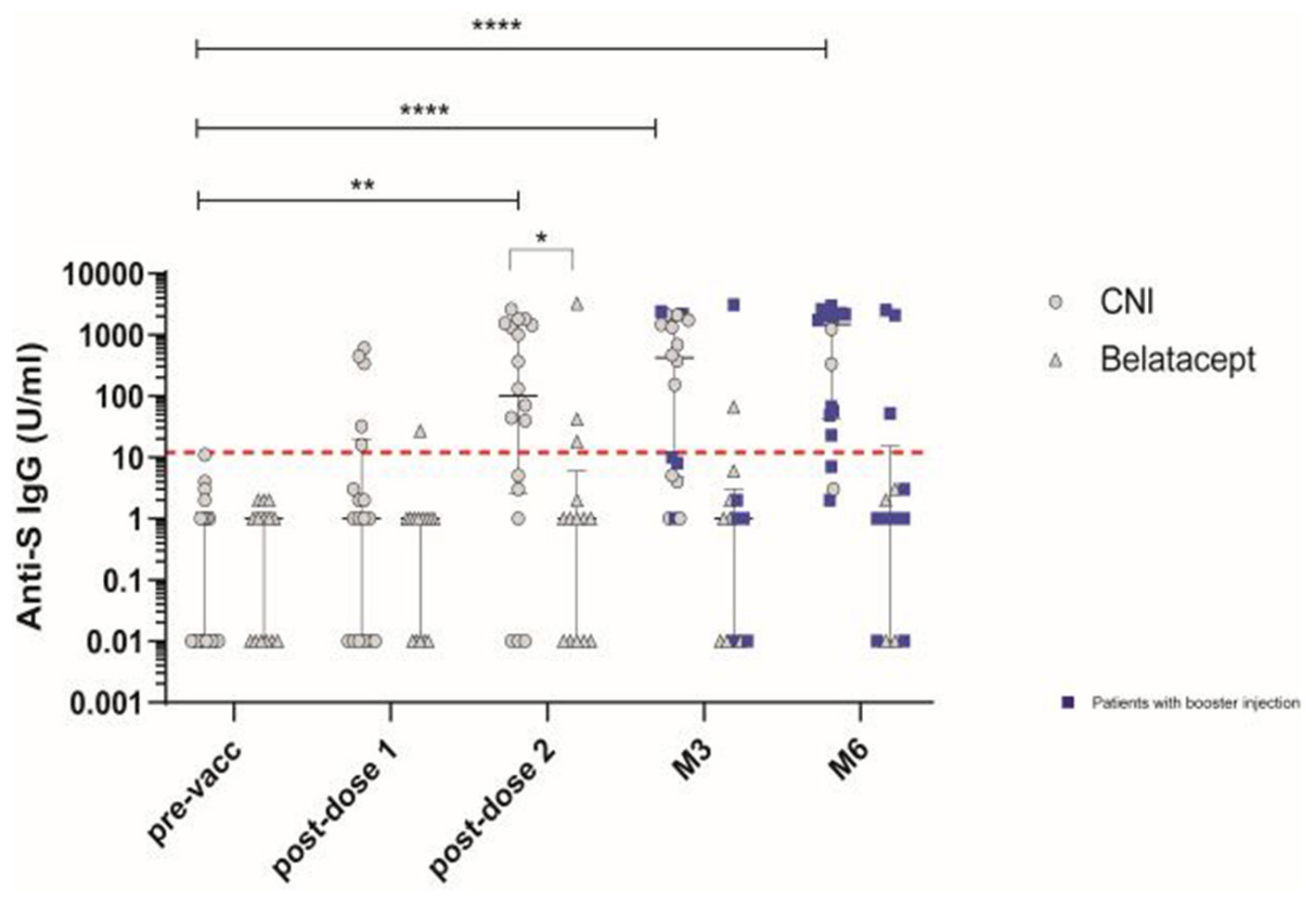
Antibody responses induced by vaccination with the BNT162b2 mRNA vaccine in patients treated with calcineurin inhibitor (CNI) or belatacept. SARS-CoV-2-specific IgG binding antibody responses directed against the native trimeric S protein at baseline (pre-vacc), 21 days after the first dose (post-dose 1), 14 days after the second dose (post-dose 2), and at M3 and M6 post-vaccination in patients treated with CNI (*n* = 18) (circles)or belatacept (*n* = 14) (triangles). Blue squares indicate patients who received the 3rd (booster) dose of vaccine at least 10 days before the follow-up visit. The dashed red line indicates the positivity threshold (12 U/ml). Median values ± IQR are shown. Mann-Whitney tests and, Friedman and Dunn's multiple comparison tests were used for statistical analysis. Statistical p value are indicated as followed (*P < 0.05, **P < 0.01, ***P < 0.001, ****P < 0.0001).

Blood samples were collected before the first vaccination (pre-vacc), 21 days after the first dose (post-dose 1), 14 days after the second dose (post-dose 2), and at M3 and M6 post-vaccination in patients treated with CNI (*n* = 18) or belatacept (*n* = 14). Two weeks after the second dose, 15/32 patients (46.9%) were tested positive for binding IgG antibodies against S. Anti-S IgG levels (median [IQR]) were significantly lower in the belatacept (1 U/ml [0.01–6]) than in the CNI group (101 U/ml [2.5–1466]); (*P* = 0.01), with 21.4% (3/14 patients) and 83.3% (15/18 patients) responders, respectively (Figure 1). KTR in the CNI group received a booster between the post-dose 2 and M3 visits (*n* = 5) or between the M3 and M6 visits (*n* = 9). The rates of responders with detectable anti-S IgG antibodies at the M3 and M6 time points in this group were 11/18 (61.1%) and 15/18 (83.3%), respectively, and all but three responders had received boosters by the M6 visit. In the belatacept group, 6/14 patients had received their boosters by the M3 visit, and 10/14 had received their booster by the M6 visit. Most KTR remained non-responders, with anti-S IgG antibody levels below the limit of detection in all but 2/14 patients (14.2%) at M3 and 3/14 patients (21.4%) at M6. All seropositive patients had received the booster by the M6 visit. Response rates differed significantly between the groups at M3 and M6 post-vaccination, with 61% and 83.3% responders at M3 and M6, respectively, in the CNI group vs. only 14.2 and 21.4%, respectively, in the belatacept group (*P* = 0.007 and *P* = 0.002 at M3 and M6, respectively; chi-squared test). Binding IgG anti-S antibody levels remained high in the CNI group at M3 and M6 (median [IQR]: 421.5 [4.7–1,812] U/ml and 1,481 [41.7–2,180] U/ml, respectively, *P* = 0.04, Wilcoxon *t*-test) (Figure 1).

**Figure 2:**
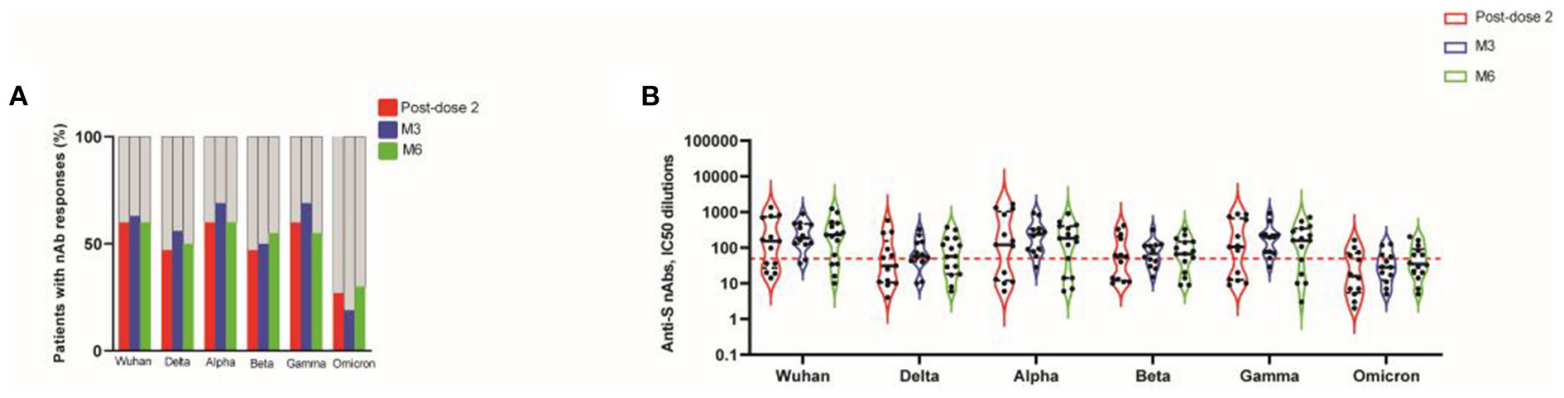
Neutralizing activity against spike protein mutations associated with VOCs, 14 days after the second dose (post-dose 2), and at M3 and M6 post-vaccination, in transplanted patients. Frequency of patients with nAb responses directed against the original strain and the various VOCs. A negative result (gray bars) indicates an IC_50_ titers < 50 dilutions; a positive result (colored bars) indicates an IC_50_ titers > 50 dilutions **(A)**. Neutralizing antibody responses were assessed by determining the half maximal inhibitory concentration (IC_50_) dilutions. The dotted red line indicates the threshold for assay positivity (i.e., IC_50_ > 50 dilutions). Data are expressed as IC_50_ (half maximal inhibitory concentration) dilutions **(B)**.

We determined nAb responses to vaccination against 2019-nCoV Wuhan and the Alpha, Beta, Gamma, Delta and Omicron variants, in a cell- and virus-free assay (6, 25, 26), in patients with detectable levels of binding IgG from the CNI group. The frequency of responders with nAb against 2019-nCoV Wuhan was 60% of KTR post-dose 2, remaining stable at M3 and M6 (Figure 2A). At M6, the response rate was 60% against Alpha, 55% against Beta, 55% against Gamma, 50% against Delta, and 30% against Omicron (Figure 2A). We evaluated the magnitude of the nAb response, using IC_50_ dilutions > 50 as the cutoff for positivity. For example, at M3, the IC_50_ titers against 2019-nCoV Wuhan, and the Delta, Alpha, Beta, Gamma and Omicron variants were (median [IQR]): 188 [125–467], 58 [41–143], 236.5 [81–336], 69.5 [33–116], 195.5 [72–233] and 28.5 [12–61], respectively. The corresponding values at M6 were 227 [38–87], 57 [18–180], 183 [14–399], 67 [20–147], 159 [18–343], and 33 [14–83]. At each time point, the IC_50_ titers against Beta, Delta and Omicron were three- to seven-fold lower than those against the Wuhan strain. By contrast, IC_50_ titers against variants remained stable throughout follow-up (Figure 2B).

**Figure 3:**
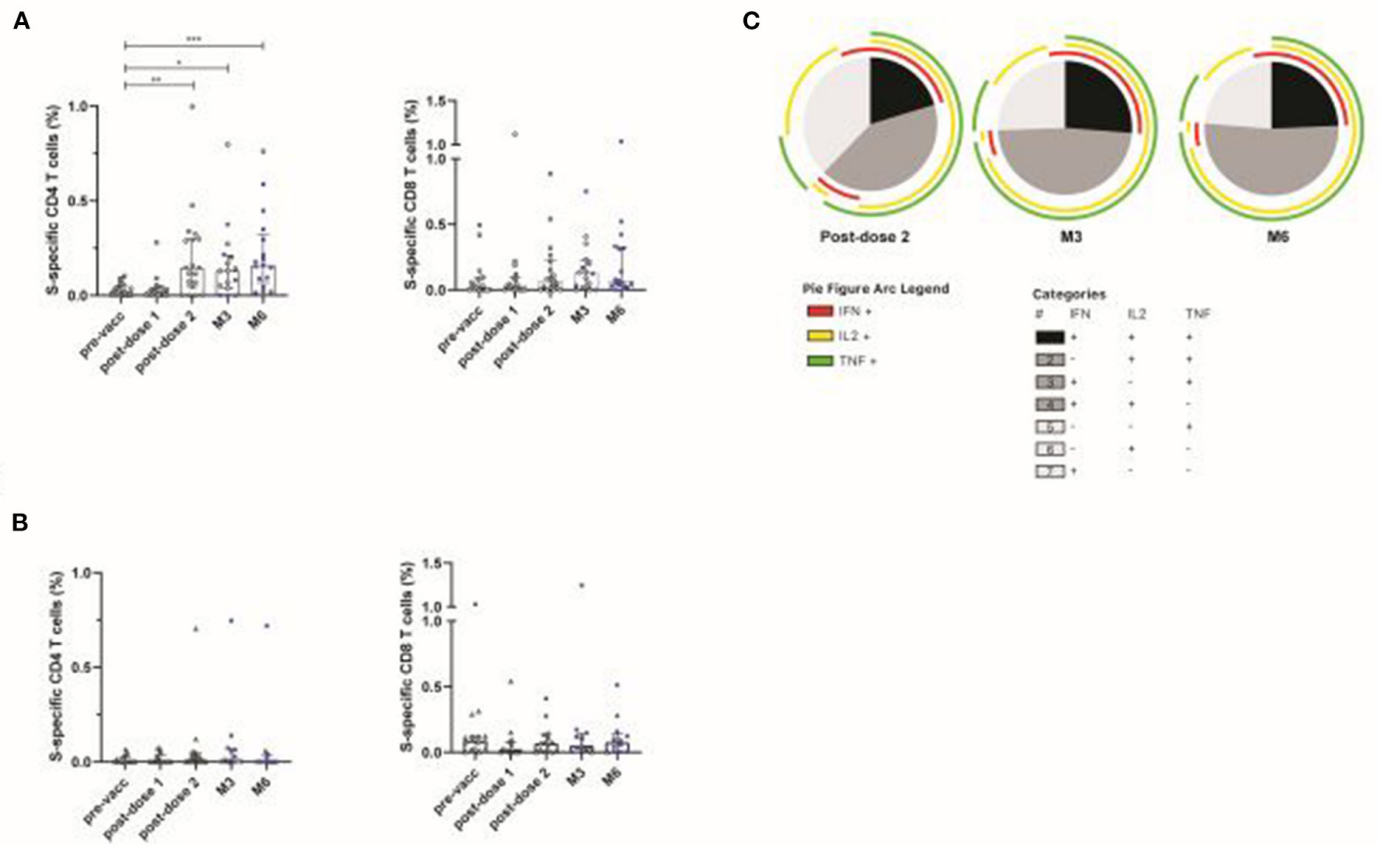
Spike-specific T-cell responses induced by vaccination with BNT162b2 in patients treated with calcineurin inhibitor (CNI) or belatacept. S-specific CD4 and CD8 T-cell responses in patients treated with CNI (*n* = 24) **(A)** or belatacept (*n* = 17) **(B)**, after overnight stimulation with a pool of overlapping peptides covering the wild-type Spike protein at baseline (pre-vacc), 21 days after the first dose (post-dose 1), 14 days after the second dose (post-dose 2), and at M3 and M6 post-vaccination. Blue squares indicate patients who received a 3rd (booster) dose of vaccine at least 10 days before the follow-up visit. Functional composition of S-specific CD4 T-cell responses in vaccinated patients treated with CNI. Responses are color-coded according to the combination of cytokines produced. The arcs identify cytokine-producing subsets (IFN-γ, IL-2, and TNF) within the CD4 T-cell population **(C)**. Kruskal-Wallis and Dunn's multiple comparison tests were used for statistical analysis (*P < 0.05, **P < 0.01, ***P < 0.001).

We then evaluated vaccine-induced T-cell responses against the Spike (S) protein, using overlapping peptide pools (OLP) spanning the S1 and S2 regions from S present in the mRNA vaccine, in KTR from the CNI (*n* = 24) and belatacept (*n* = 17) groups. After two or three doses of vaccine, the median frequencies [IQR] of S-specific CD4 T cells (IFN-γ ± IL-2 ± TNF) in CNI-treated patients were 0.15% [0.04–0.3]; 0.13% [0.04–0.21] and 0.16% [0.07–0.8], at 15 days post-dose 2, M3 and M6, respectively (*P* = 0.009; *P* = 0.04, and *P* = 0.0007 vs. the pre-vaccination visit). A longitudinal analysis of long-term follow-up visits (M3 and M6) showed no decrease in the frequency of S-specific T-cell responses relative to the post-dose 2 time point. Several patients received boosters (third dose) during this period, which may account for the maintenance of the cellular response. No increase in S-specific CD8 T cells was detected after vaccination with the BNT162b2 mRNA vaccine (Figure 3A). By contrast, no S-specific CD4 or CD8 T cells were detected in patients treated with belatacept, as shown by comparison with the pre-vaccination visit (Figure 3B). S-specific CD4 T-cell responses in the CNI group were polyfunctional, with the simultaneous production of up to three cytokines, and no major differences from post-dose 2 to M6 (Figure 3C). Thus, KTR treated with belatacept are less likely to be capable of mounting humoral and T-cell responses after the two initial injections or booster vaccination.

Globally, KTR remained highly susceptible to breakthrough infection. Fifteen (8 in the CNI group, corresponding to 24.2%; and 7 in the belatacept group, corresponding to 33.3%) of the 52 patients developed symptomatic COVID-19 during follow-up. One fatal case occurred a few days after vaccination in the CNI group.

## Discussion

Since the beginning of the SARS-CoV-2 pandemic, immunocompromised individuals have remained a fragile population highly susceptible to severe disease and with a weak response to current vaccines or following natural infection (27). The humoral immune response (either nAb or non-nAbs) following natural infection or vaccination has been shown to be protective (28–31). In immunocompetent individuals, vaccine-elicited anti-S antibodies are detected in up to 95% of those vaccinated with mRNA vaccines (32). Antibody detection rates after vaccination are much lower in solid organ transplant (SOT) patients on long-term maintenance immunosuppressive therapy (3, 6). This study significantly extends these previous findings, by showing that both humoral and cellular responses are weaker in KTR, even after booster vaccination. Humoral responses in our group of kidney transplant patients were weaker than those measured with the same assays in a historical cohort of healthy volunteers (median: 1,900.4 U/ml; 95% CI, 1,816.1–2,119.8) (6). We also show that belatacept, a checkpoint inhibitor of immune cell activation, almost completely abolishes the ability of patients to mount a vaccine response. This observation has significant clinical implications, because these patients should undergo a careful evaluation of their vaccine responses and should benefit from prophylactic measures. Overall, our results are consistent with previous studies showing a weak response to SARS-CoV-2 vaccination in solid organ transplant patients (3, 16). However, we also demonstrate that belatacept-treated patients have much lower response rates than patients treated with CNI, indicating a strong dependence of the vaccine responses of these patients on the mechanism of action of their immunosuppressive regimens (18, 19). These results are consistent with those of the BENEFIT and BENEFIT-Ext studies, showing that patients treated with belatacept are significantly less likely to develop *de novo* anti-donor anti-HLA antibodies than patients on a CNI-based regimen (20). Thus, belatacept is more effective at preventing *de novo* B-cell activation than CNI, even though CNI strongly inhibit T-cell activation and the cytokine production (IL-2) required for B-cell activation (33–35).

T cells are also crucial for viral clearance (36). SARS-CoV-2 virus clearance has been reported in patients with Bruton agammaglobulinemia (37). Specific T-cell functionality increased after booster vaccination in CNI-treated patients but not in belatacept-treated patients, suggesting that belatacept-treated patients are at risk of severe COVID or long-term infection due to a lack of both T- and B-cell responses. This is in agreement with the observations of Zhang et al. who found an impaired CD4 T cell response in tacrolimus or belatacept treated patients (38). In addition, the development of a specific high-affinity immune response requires the coordination of T and B cells, CD4 follicular T cells (Tfhs) being essential for the maturation and differentiation of B cells for the synthesis of high-affinity antibodies (36). We show here that T-cell responses are not homogeneous and can be severely impaired in SOT patients, as reported for immunocompromised patients (6, 16). On the contrary to Zhang's study, this effect was more marked for belatacept-treated patients, consistent with previous studies showing that belatacept impairs the naïve T-cell response and B-cell help, with a lesser effect on memory T cells (21, 38–41). Thus, belatacept, which inhibits the CD28 pathway in addition to its direct effect on B-cell maturation, inducing the accumulation of transitional B cells (42), also impairs T-cell activation after vaccination. As a result, anti-S IgG responses were almost undetectable in most patients treated with belatacept. We also show, despite their ability to inhibit T-cell activation and IL-2 production, CNI have a much milder effect on humoral responses. In CNI-treated patients, the humoral response may be preserved to some extent through extragerminal center cross-reacting B lymphocytes, as recently reported (43). These cross-reacting B cells, and the impairment of CD4^+^ T-cell help may contribute to the steeper slope of the antibody level curve in these patients (6, 43).

Clinically, these results highlight the need to check the biological immune response to vaccination in immunocompromised patients, particularly those treated with belatacept, which almost completely abolishes humoral responses in most patients. No benefit of an additional booster injection would be expected in patients on belatacept, whereas an improvement of humoral responses was observed in CNI-treated patients. However, all these patients remain at high risk of infection, because vaccine responders display significantly lower levels of neutralizing antibody activity against VOC than the general population, with lower levels of activity against the Omicron variant (6) than for other variants, as reported in the general population. Thus, for immunocompromised patients with low titers of anti-S antibodies, additional prophylactic or post-exposure strategies may be required in cases of infection.

In accordance with national recommendations, the patients from our belatacept group were offered treatment with anti-SARS-CoV-2 monoclonal antibodies (*n* = 9) for the prevention (*n* = 8) or treatment (*n* = 1) of infection. One patient also received high-titer convalescent plasma. Of note, these treatments were administered after our study. However, unsurprisingly, given the epidemiology of the emergence of more infectious variants, a number of breakthrough infections (BI) were observed in our cohort. Mortality rates were high during the first and second waves of the pandemic, but only two deaths occurred in this cohort. One death, related to BI, occurred shortly after the first dose of vaccine was administered, suggesting that the patient did not develop an effective immune response.

One of the limitations of this study is the small number of patients that we were able to include, due to the need to vaccinate as many patients as possible, as rapidly as possible, and frequently close to their site of residence, outside of hospital structures. This also affected the availability of samples for exhaustive T- and B-cell assessments.

Overall, our results confirm the weak antibody and cellular responses of transplant recipients vaccinated with BNT162b2, particularly those treated with belatacept. New vaccination strategies are required for these patients, possibly including immunosuppression conversion under close monitoring for a limited period around vaccination, given the risk of allogeneic immunization, to facilitate the development of a vaccine response.

## Data availability statement

The original contributions presented in the study are included in the article/supplementary material, further inquiries can be directed to the corresponding author.

## Ethics statement

The studies involving human participants were reviewed and approved by Ethics Committee (No. IDRCB: 2018-A01610-55). The patients/participants provided their written informed consent to participate in this study.

## Author contributions

YL, AD, and AW conceived and designed the study. AW, CP, CL, GP, AD, and YL analyzed and interpreted the data. LG, MDé, CK, and CP performed the experiments. AD, MDe, and PG participated in sample and clinical data collection. AW, CP, AD, and YL drafted the first version and wrote the final version of the manuscript. All authors approved the final version.

## Funding

This work was supported by INSERM and the Investissements d'Avenir program, and the Vaccine Research Institute (VRI), managed by the ANR under reference ANR-10-LABX-77-01 and by the European Union's Horizon Europe Research and Innovation program under Grant Agreement No. 101046041.

## Conflict of interest

The authors declare that the research was conducted in the absence of any commercial or financial relationships that could be construed as a potential conflict of interest.

## Publisher's note

All claims expressed in this article are solely those of the authors and do not necessarily represent those of their affiliated organizations, or those of the publisher, the editors and the reviewers. Any product that may be evaluated in this article, or claim that may be made by its manufacturer, is not guaranteed or endorsed by the publisher.

## Author disclaimer

The views and opinions expressed are those of the authors only, and do not necessarily reflect those of the European Union or the European Commission.
